# *Mycoplasma hominis* brain abscess presenting after a head trauma: a case report

**DOI:** 10.1186/1752-1947-6-253

**Published:** 2012-08-22

**Authors:** Andrés F Henao-Martínez, Heather Young, Johanna Jacoba Loes Nardi-Korver, William Burman

**Affiliations:** 1 Department of Medicine, Division of Infectious Diseases, 12700 E. 19th Avenue, Mail Stop B168, Aurora, CO 80045, USA; 2 Department of Pathology, University of Colorado Denver, 12700 E. 19th Avenue, Mail Stop B168, Aurora, CO 80045, USA

## Abstract

**Introduction:**

*Mycoplasma hominis* brain abscess is a rare occurrence, and treatment is not well defined. The mechanism by which *M. hominis* infects sites outside the genitourinary tract, including the central nervous system, is unclear.

**Case presentation:**

We report the case of a 40-year-old Somali man who sustained a traumatic brain injury that required initial neurosurgical hematoma evacuation and that subsequently was complicated by a hospital-acquired *M. hominis* brain abscess. Our patient was successfully treated with neurosurgical debridement and an antibiotic course of intravenous doxycycline.

**Conclusions:**

Head trauma or neurosurgical procedures or both might be a predisposing factor for this type of infection.

## Introduction

*Mycoplasma hominis* is generally a genitourinary (GU) pathogen. *M. hominis* brain abscess is rare. To the best of our knowledge, only ten cases have been reported in the literature. However, the true incidence may be higher than this because the organism is difficult to grow *in vitro*. Optimal treatment of *M. hominis* brain abscess is not well defined in the literature. The organism is uniformly resistant to macrolides, and other potentially useful agents do not penetrate the central nervous system well.

## Case presentation

**Figure 1:**
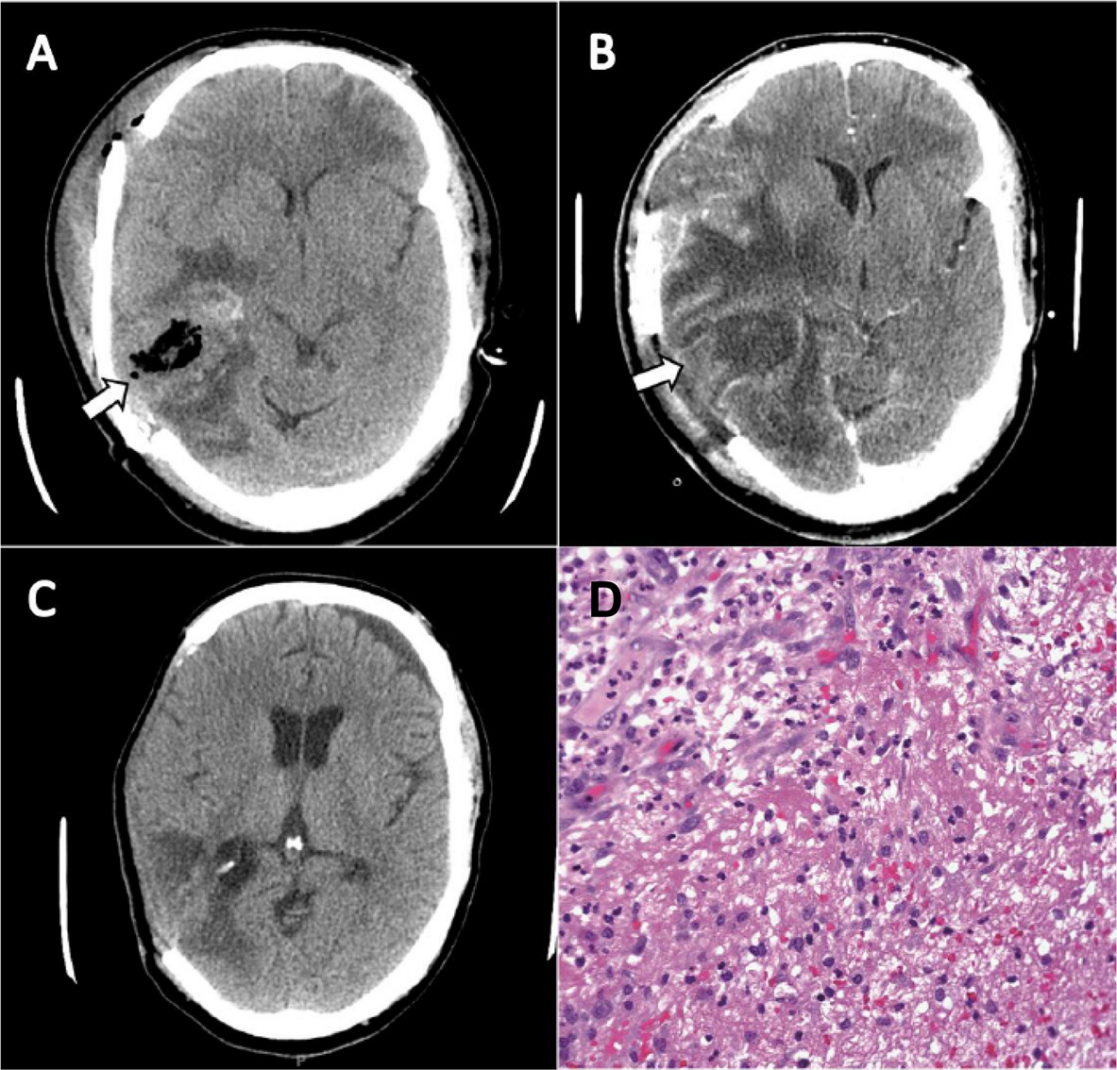
**CT images and Pathology description:** (**a**) Multiple foci of gas-containing abscesses along the margin of the craniotomy (arrow). (**b**) Significant increase in the size and extent of the brain abscess (arrow). (**c**) Resolving brain swelling and abscess. (**d**) Cerebral abscess in the superior temporal lobe with necrosis, newly formed capillaries, and scattered fibroblasts. The inflammatory infiltrate includes neutrophils, lymphocytes, plasma cells, and histiocytes (stain: hematoxylin and eosin; magnification: ×400).

A previously healthy 40-year-old Somali man presented after a motor vehicle accident. Injuries included a right subdural hematoma, subarachnoid hemorrhages, intra-parenchymal contusions, facial fractures, and a left humerus fracture. The subdural hematoma was evacuated by means of a craniotomy on hospital day 7. On hospital day 13, our patient developed a fever of 39. 2 °C and purulent drainage from the craniotomy wound. A computed tomography (CT) scan of his head without intravenous contrast demonstrated multiple foci of gas-containing abscesses along the margin of the craniotomy (Figure 1a). The air in the abscess was considered part of the expected post-craniotomy changes. Incision, drainage, and removal of the bone flap were promptly performed. Vancomycin and piperacillin/tazobactam were initiated and then narrowed to ceftriaxone and metronidazole when a Gram stain revealed many polymononuclear cells but no organisms. Despite this management, fevers continued and our patient’s mental status failed to improve. A CT scan of his head without intravenous contrast on hospital day 20 revealed an increase in both the size and extent of the brain abscess (Figure 1a). Our patient underwent a second debridement; again a Gram stain revealed no organisms. A pathology examination of the brain abscess showed a cerebral abscess in the superior temporal lobe with necrosis but without organisms (Figure 1d).

Cultures on sheep’s blood agar from the neurosurgical debridements revealed small colonies with a peripheral clearing (‘fried egg’ morphology). Through sequencing of the 16 S ribosomal subunit region at a reference laboratory, the isolate was identified as *M. hominis* with 100% homology to reference strains. Our patient’s antibiotics were changed to doxycycline 100mg intravenously twice per day on hospital day 24. His mental status and fevers improved significantly. The abscess had almost fully resolved by hospital day 40 (Figure 1c).

## Discussion

*M. hominis* is traditionally considered to be a GU pathogen and has been implicated in non-gonococcal urethritis and post-partum fevers and is a possible cause of bacterial vaginosis [1,2]. In addition to causing infection, *M. hominis* is well known to colonize the GU and respiratory tracts [3-5]. *M. hominis* infections outside the GU tract are uncommon, but the organism has been isolated in cases of bacteremia, inguinal wound infections, sternotomy infection, septic prosthetic hip arthritis, pleural empyema, ventriculo-peritoneal shunt infections, and post-neurosurgical wound infections [6].

Eleven cases of *M. hominis* brain abscess [7-15], including this case, have been published (Table 1). Eight of these cases were related to head trauma or craniotomy. The mean time to *M. hominis* isolation in the published literature is 13.5 days, suggesting that patients acquire *M. hominis* brain abscess while in the hospital. Hemorrhagic lesions in the brain could be seeded by contiguous infection after disruption of a colonized upper airway following trauma or after transient bacteremia from manipulation of the colonized oropharyngeal or GU tracts or both.

**Table 1:** Cases of adult Mycoplasma hominis brain abscess

**Case**	**Year**	**Age and sex**	**Associated condition**	**First culture after AD**	**Therapy**
1	1981	29-year-old man	Head trauma	21 days	Tetracycline
2	1995	20-year-old man	Head trauma	14 days	Cefotaxime + metronidazole + doxycycline
3	1997	22-year-old woman	Post-partum	8 days	Ceftriaxone + metronidazole
4	2002	40-year-old woman	Cavernous angioma/craniotomy	4 days	Ciprofloxacin + metronidazole
5	2003	17-year-old girl	Post-partum	13 days	Doxycycline + clindamycin
6	2004	40-year-old man	Head trauma	14 days	Tetracycline
7	2008	17-year-old girl	Head trauma	17 days	Gatifloxacin
8	2008	48-year-old man	Craniotomy for colloid cyst removal	18 days	Gatifloxacin + clindamycin
9	2003/ 2006	NA	Post-traumatic	NA	Doxycycline
10^a^	2011	40-year-old man	Head trauma	13 days	Doxycycline

^a^Present report. *AD*: admission day; *NA*: not available.

*M. hominis* is uniformly resistant to macrolides. Fluoroquinolones, tetracyclines, and clindamycin have better *in vitro* antimicrobial activity against *M. hominis *[16,17]. These three classes of antibiotics possess similar, moderate rates of blood–brain barrier penetration [18]. Rates of *in vitro* resistance to these antibiotics may be as high as 10% for clindamycin, 27% for doxycycline, and 80% for ciprofloxacin. However, there have been successful reports for each of these therapies.

## Conclusions

*M. hominis* brain abscess is rare. The mechanism of *M. hominis* brain abscess is likely to be either direct spread from a colonized oropharynx to an open head wound or seeding of an intra-cranial hematoma from transient bacteremia after manipulation of the GU tract. *M. hominis* brain abscesses are treatable with surgical drainage and appropriate antimicrobial therapy of a tetracycline, clindamycin, or fluoroquinolone.

## Consent

Written informed consent was obtained from the patient for publication of this case report and accompanying images. A copy of the written consent is available for review by the Editor-in-Chief of this journal.

## Abbreviations

CT, computed tomography; GU, genitourinary.

## Competing interests

The authors declare that they have no competing interests.

## Authors’ contributions

AFH-M was the major contributor in studying the case and writing the manuscript and was involved in the medical care of the patient. HY contributed to the writing and editing of the manuscript. JJLN-K is on the faculty of the Department of Pathology and was responsible for the brain biopsy reading. WB is on the faculty of the Division of Infectious Diseases, is the director of Denver Public Health, and was involved in the manuscript editing and in the medical care of the patient. All authors read and approved the final manuscript.
